# Vitamin A5/X, a New Food to Lipid Hormone Concept for a Nutritional Ligand to Control RXR-Mediated Signaling

**DOI:** 10.3390/nu13030925

**Published:** 2021-03-12

**Authors:** Wojciech Krężel, Aurea Rivas, Monika Szklenar, Marion Ciancia, Rosana Alvarez, Angel R. de Lera, Ralph Rühl

**Affiliations:** 1 Institut de Génétique et de Biologie Moléculaire et Cellulaire (IGBMC), 67404 Illkirch, France; ciancia@igbmc.fr; 2 Inserm, U 1258, 67404 Illkirch, France; 3 CNRS UMR 7104, 67404 Illkirch, France; 4 Université de Strasbourg, 67404 Illkirch, France; 5 Departamento de Química Orgánica, Facultade de Química, CINBIO and IBIV, Universidade de Vigo, Campus As Lagoas-Marcosende, 36310 Vigo, Spain; krsezel@igbmc.fr (A.R.); rar@uvigo.es (R.A.); 6 Paprika Bioanalytics BT, 4002 Debrecen, Hungary; monikaszklenar1@gmail.com; 7 CISCAREX UG, 13351 Berlin, Germany

**Keywords:** vitamin A, retinoid, retinoic acid, memory, cognitive function, carotene, carotenoid

## Abstract

Vitamin A is a family of derivatives synthesized from carotenoids acquired from the diet and can be converted in animals to bioactive forms essential for life. Vitamin A1 (all-*trans*-retinol/ATROL) and provitamin A1 (all-*trans*-β,β-carotene/ATBC) are precursors of all-*trans*-retinoic acid acting as a ligand for the retinoic acid receptors. The contribution of ATROL and ATBC to formation of 9-*cis*-13,14-dihydroretinoic acid (9CDHRA), the only endogenous retinoid acting as retinoid X receptor (RXR) ligand, remains unknown. To address this point novel and already known retinoids and carotenoids were stereoselectively synthesized and administered *in vitro* to oligodendrocyte cell culture and supplemented *in vivo* (orally) to mice with a following high-performance liquid chromatography-mass spectrometry (HPLC-MS)/UV-Vis based metabolic profiling. In this study, we show that ATROL and ATBC are at best only weak and non-selective precursors of 9CDHRA. Instead, we identify 9-*cis*-13,14-dihydroretinol (9CDHROL) and 9-*cis*-13,14-dihydro-β,β-carotene (9CDHBC) as novel direct nutritional precursors of 9CDHRA, which are present endogenously in humans and the human food chain matrix. Furthermore, 9CDHROL displayed RXR-dependent promnemonic activity in working memory test similar to that reported for 9CDHRA. We also propose that the endogenous carotenoid 9-*cis*-β,β-carotene (9CBC) can act as weak, indirect precursor of 9CDHRA via hydrogenation to 9CDHBC and further metabolism to 9CDHROL and/or 9CDHRA. In summary, since classical vitamin A1 is not an efficient 9CDHRA precursor, we conclude that this group of molecules constitutes a new class of vitamin or a new independent member of the vitamin A family, named “Vitamin A5/X”.

## 1. Introduction

Retinoid X receptors (RXRα, β and γ) are nuclear hormone receptors involved in the regulation of various signaling pathways relevant for control of lipid metabolism, inflammation, proliferation and differentiation, controlling thereby functions of diverse organs including the brain [1]. Accordingly, RXR-related signaling has been reported to be dysregulated in various diseases ranging from cancer, neurological, cardiovascular or skin/immune-related disorders [2]. Heterodimerization of RXRs with other nuclear hormone receptors directly underlies such pleiotropic functions of RXRs [3,4]. Importantly, signaling of RXR homodimers but also heterodimers with some nuclear receptors, including for example liver X receptors (LXRs), peroxisome proliferator-activated receptors (PPARs) or nuclear receptor subfamily 4 group A (NR4A), is regulated by RXR ligands [5]. 9-*cis-*Retinoic acid (9CRA) originating from a simple isomerization of all-*trans*-retinoic acid (ATRA), known as the major endogenous, physiologically relevant RAR-ligand [6,7], was for a long time considered as the endogenous ligand for RXR-activation [8,9]. This theory was and still is fragile, remains controversial and was never generally and completely proven in mammals [10,11,12,13].

Recently, 9-*cis*-13,14-dihydroretinoic acid (9CDHRA) has been identified as an endogenous retinoid and an RXR ligand with physiological relevance in mice [10] opening thus the possibility of dynamic, ligand-dependent control of RXR-related functions and pathologies. In mice, reduced available levels of 9CDHRA have been associated with spatial working memory deficits, shown previously to be highly sensitive to ligand-dependent functions of RXR [11]. Accordingly, pharmacological treatment with 9CDHRA displayed memory-enhancing effects in delayed spatial tasks in mice. Such findings point to the possibility that limited availability of 9CDHRA is most likely related to nutritional intake of its precursor(s) or dysfunction of precursor metabolism. This may compromise ligand-dependent functions of RXRs and lead to some of the RXR-related pathologies, which span beyond mnemonic deficits [14,15,16,17]. Critical for addressing such a hypothesis is knowledge of the precursor(s) and metabolic pathway(s) involved in generation of 9CDHRA, which remain virtually unexplored. Whereas a previous study indicated an involvement of retinol binding protein, Rbp1, as an important component of the metabolic pathway for 9CDHRA synthesis [11], the biologically-relevant precursors are not known, but have been conceptualized [12]. Thus, specific isomerization of the 9-*cis*-double bond (reviewed in [10,12]) and selective saturation of the C13,C14-double bond [18,19,20] have been proposed as potential direct and indirect pathways for the conversion of all-*trans*-retinol (ATROL), known as vitamin A1, to dihydroretinoids including 9CDHRA. 

Indeed, ATROL and all-*trans*-β,β-carotene (ATBC) could be considered as the most likely precursors of 9CDHRA. If such a hypothesis were validated, 9CDHRA could be considered as a new active metabolite of ATROL and a member of the vitamin A1 cluster. Otherwise, 9CDHRA should be considered as a member of a new class of vitamin or at least a new class of the vitamin A family. In this study we focused on the identification of physiologically- and nutritionally-relevant precursor(s) of the RXR-ligand, 9CDHRA. 

## 2. Materials and Methods

### 2.1. Organic Chemical Synthesis

#### 2.1.1. *Synthesis of (R)-9-cis-13,14-Dihydroretinol **(R)-(2)** (9CDHROL) and of the Corresponding Acetate **(R)-(3)***

**Figure 1:**
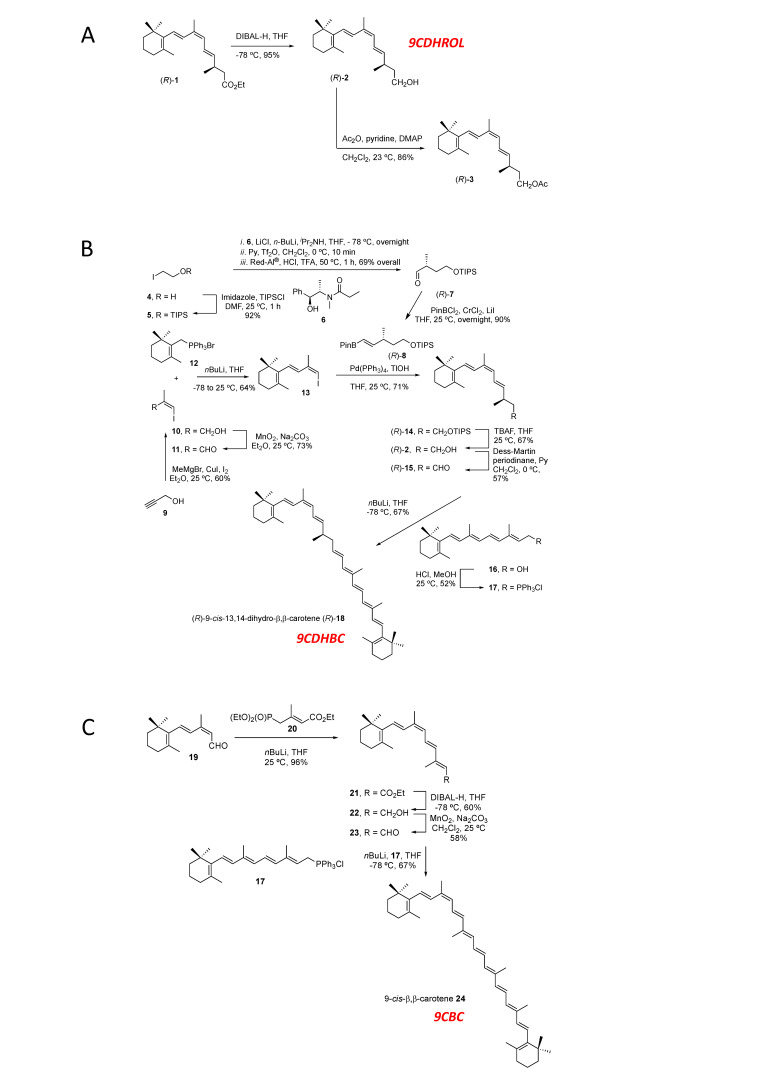
**Synthesis of vitamin A5/X and provitamin A5/X.** (**A**): Synthesis of (*R*)-9-*cis*-13,14-dihydroretinol (9CDHROL), **(*R*)-(2)** and its acetate **(*R*)-(3)**; (**B**). Synthesis of (*R*)-9-*cis*-13,14-dihydro-β,β-carotene (9CDHBC), **(*R*)-(18)**; (**C**). Synthesis of 9-*cis*-β,β-carotene (9CBC) **(24)**.

(*R*)-9-*cis*-13,14-dihydroretinol **(*R*)-(2)** was synthesized by DIBAL-H reduction of the already described ethyl (*R*)-9-*cis*-13,14-dihydroretinoate **(*R*)-(1)** [11] in THF at −78 °C in 95% yield. (*R*)-9-*cis*-13,14-dihydroretinyl acetate **(*R*)-(3**) was prepared in 86% yield by acetylation of (*R*)-9-*cis*-13,14-dihydroretinol **(*R*)-(2)** with acetic anhydride and pyridine in the presence of dimethyl-amino-pyridine (DMAP) (Figure 1A).

(3*R*,4*E*,6*Z*,8*E*)-3,7-Dimethyl-9-(2,6,6-trimethylcyclohex-1-en-1-yl)nona-4,6,8-trien-1-ol **(*R*)-(2)**: To a cooled (−78 °C) solution of ethyl (3*R*,4*E*,6*Z*,8*E*)-3,7-dimethyl-9-(2,6,6-trimethylcyclohex-1-en-1-yl)nona-4,6,8-trienoate **(*R*)-(1)** (ethyl (*R*)-9-*cis*-13,14-dihydroretinoate) (32.0 mg, 0.097 mmol) in THF (1.0 mL), DIBAL-H (0.242 mL, 0.242 mmol, 1.0 M in hexanes) was added and the resulting mixture was stirred for 30 min at −78 °C. The mixture was allowed to warm up to −20 °C in 2.5 h. H_2_O was added and the mixture was extracted with Et_2_O (3x). The combined organic layers were dried (Na_2_SO_4_) and the solvent removed. Flash chromatography of the residue (silica gel, first neutralized with 98:2 hexane/Et_3_N, then gradient from 95:5 hexane/EtOAc to EtOAc) afforded (3*R*,4*E*,6*Z*,8*E*)-3,7-dimethyl-9-(2,6,6-trimethylcyclohex-1-en-1-yl)nona-4,6,8-trien-1-ol **(*R*)-(2)** (26.5 mg, 95%) as a colorless oil. ^1^H-NMR (400.13 MHz, C_6_D_6_): δ 6.93 (d, *J* = 16.0 Hz, 1H), 6.69 (dd, *J* = 14.9, 11.1 Hz, 1H), 6.29 (d, *J* = 16.0 Hz, 1H), 6.01 (d, *J* = 11.1 Hz, 1H), 5.46 (dd, *J* = 15.0, 8.3 Hz, 1H), 3.35 (t, *J* = 6.6 Hz, 2H), 2.27 (dt, *J* = 14.0, 6.7 Hz, 1H), 1.98–1.92 (m, 2H), 1.93 (s, 3H), 1.79 (s, 3H), 1.63–1.52 (m, 2H), 1.51–1.43 (m, 2H), 1.36 (q, *J* = 6.7 Hz, 2H), 1.11 (s, 6H), 0.91 (d, *J* = 6.7 Hz, 3H) ppm. ^13^C-NMR (100.62 MHz, C_6_D_6_): δ 140.0 (d), 138.6 (s), 132.5 (s), 130.9 (d), 129.6 (d), 129.2 (s), 128.1 (d), 124.9 (d), 60.9 (t), 40.2 (t), 39.9 (t), 34.5 (s), 34.3 (d), 33.2 (t), 29.21 (q), 29.18 (q), 22.0 (q), 20.9 (q), 20.7 (q), 19.7 (t) ppm.

(3*R*,4*E*,6*Z*,8*E*)-3,7-Dimethyl-9-(2,6,6-trimethylcyclohex-1-en-1-yl)nona-4,6,8-trien-1-yl Acetate **(*R*)-(3)**: To a solution of (3*R*,4*E*,6*Z*,8*E*)-3,7-dimethyl-9-(2,6,6-trimethylcyclohex-1-en-1-yl)nona-4,6,8-trien-1-ol **(*R*)-(2)** (5.7 mg, 0.020 mmol) in CH_2_Cl_2_ (0.5 mL) were sequentially added Ac_2_O (0.009 mL, 0.099 mmol), pyridine (0.008 mL, 0.099 mmol) and DMAP (0.5 mg, 0.004 mmol). The resulting mixture was stirred for 1 h at 25 °C. Then, Et_2_O was added and the resulting solution was washed with a saturated aqueous solution of CuSO_4_ (2x). The organic layer was dried (Na_2_SO_4_) and evaporated. Flash chromatography of the residue (silica gel, gradient from hexane to 85:15 hexane/EtOAc) afforded (3*R*,4*E*,6*Z*,8*E*)-3,7-dimethyl-9-(2,6,6-trimethylcyclohex-1-en-1-yl)nona-4,6,8-trien-1-yl acetate **(*R*)-(3)** (5.6 mg, 86%) as a colorless oil. ^1^H-NMR (400.13 MHz, C_6_D_6_): δ 6.92 (d, *J* = 16.0 Hz, 1H), 6.67 (dd, *J* = 15.0, 10.8 Hz, 1H), 6.29 (d, *J* = 16.0 Hz, 1H), 5.98 (d, *J* = 11.1 Hz, 1H), 5.39 (dd, *J* = 15.0, 8.2 Hz, 1H), 4.11–3.92 (m, 2H), 2.16 (dq, *J* = 14.4, 7.2 Hz, 1H), 1.99–1.90 (m, 2H), 1.93 (s, 3H), 1.79 (d, *J* = 1.0 Hz, 3H), 1.67 (s, 3H), 1.62–1.53 (m, 2H), 1.50–1.41 (m, 4H), 1.11 (s, 6H), 0.86 (d, J = 6.8 Hz, 3H) ppm. 

#### 2.1.2. *Synthesis of (R)-9-cis-13,14-Dihydro-β,β-Carotene (9CDHBC; Figure 1B, including Numbering)*

(2-Iodoethoxy)-triisopropylsilane **(5)**. To a cooled (0 °C) solution of 2-iodoethanol **(4)** (2 g, 11.63 mmol) in DMF (31.4 mL) were sequentially added imidazole (1.27 g, 18.61 mmol) and *^i^*Pr_3_SiCl (3.2 mL, 15.12 mmol). After stirring for 1 h at 25 °C, the reaction was quenched with a saturated aqueous solution of NaCl and extracted with Et_2_O (3x). The combined organic layers were washed with H_2_O (3x) and dried (Na_2_SO_4_) and the solvent was evaporated. The residue was purified by column chromatography (silica gel, 98:2 hexane/EtOAc) to afford 2.33 g (92%) of a yellow oil identified as (2-iodoethoxy)-triisopropylsilane 5. ^1^H-NMR (400.16 MHz, CDCl_3_): δ 3.91 (t, 2H, 2H_1_), 3.22 (t, 2H, 2H_2_), 1.07–1.01 (m, 21H, SiCH(CH_3_)_2_) ppm. ^13^C-NMR (101.63 MHz, CDCl_3_): δ 64.7 (t), 18.1 (1, 6x), 12.1 (d, 3x), 6.9 (t) ppm. IR (NaCl): ν 2943 (m, C–H), 2865 (m, C–H), 1462 (m), 1379 (m), 1094 (m), 881 (m) cm^−1^. HRMS (ESI^+^): Calcd. for C_11_H_26_IOSi ([M+H]^+^), 329.0790; found, 329.0792. 

(*R*)-2-Methyl-4-((triisopropylsilyl)oxy)-butanal **(*R*)-(7)**. To a cooled (0 °C) suspension of anhydrous LiCl (5.42 g, 127.93 mmol) in THF (56.1 mL) and *N*,*N*-diisopropylamine (9 mL, 63.97 mmol) was added *n*-BuLi (23.9 mL, 2.5 M in hexanes, 59.7 mmol) and the mixture was stirred for 15 min at 0 °C and for 20 min at 25 °C. The mixture was cooled down to −78 °C, a solution of *N*-((2*R*,3*R*)-3-hydroxy-3-phenylpropan-2-yl)-*N*-methylpropionamide **(6)** (6.6 g, 29.85 mmol) in THF (96.9 mL) was added and the resulting reaction mixture was stirred for 45 min at −78 °C, 15 min at 0 °C and 15 min at 25 °C. After cooling down to −78 °C, (2-iodoethoxy)-tri-isopropyl-silane **(5)** (7 g, 21.32 mmol) was added and the resulting mixture was stirred for 17 h at 0 °C. The reaction mixture was quenched with a saturated aqueous solution of NH_4_Cl and extracted with EtOAc (3x). The combined organic layers were washed with brine (3x) and dried (Na_2_SO_4_) and the solvent was evaporated. The residue was purified by column chromatography (silica gel, 80:20 hexane/EtOAc) to afford 7.5 g (84%) of a yellow oil, which was used in the next step. To a cooled (0 °C) solution of the compound obtained above (7.5 g, 17.87 mmol) in THF (128 mL), Red-Al^®^ (11 mL, 3.5 M in toluene, 26.8 mmol) was added. After stirring for 20 min, TFA (1.4 mL, 17.87 mmol) and HCl (1.8 mL, 1 M) were added. The reaction mixture was stirred for 1 h at 50 °C. The resulting mixture was then cooled down to 25 °C, Et_2_O and a 1M HCl solution (50 mL, 1:1 *v*/*v*) were added and the mixture was extracted with Et_2_O (3x). The combined organic layers were washed with an aqueous solution of NaHCO_3_ (1x), dried (Na_2_SO_4_) and the solvent was evaporated. The residue was purified by column chromatography (silica gel, 90:10 hexane/EtOAc) to afford 3.2 g (69%) of a yellow oil identified as (*R*)-2-methyl-4-((triisopropylsilyl)oxy)-butanal **(7)**. [α]_D_^17^ −1.81° (*c* 0.61, MeOH). ^1^H-NMR (400.16 MHz, C_6_D_6_): δ 9.42 (s, 1H, H_1_), 3.67–3.39 (m, 2H, 2H_4_), 2.30–2.10 (m, 1H, H_2_), 1.79–1.67 (m, 1H, H_3A_), 1.37–1.25 (m, 1H, H_3B_), 1.06–1.04 (m, 21H, SiCH(CH_3_)_2_), 0.84 (d, *J* = 7.1 Hz, 3H, C2-CH_3_) ppm. ^13^C-NMR (101.63 MHz, C_6_D_6_): δ 203.2 (d), 61.0 (t), 43.6 (d), 34.1 (t), 18.3 (q, 6x), 18.1 (d, 3x), 12.3 (q) ppm. IR (NaCl): ν 2940 (m, C–H), 2867 (m, C–H), 2717 (m), 1727 (s, C=O), 1462 (m), 1463 (m), 1105 (m), 883 (m) cm^−1^. HRMS (ESI^+^): Calcd. for C_14_H_31_IOSi ([M+H]^+^), 259.2087; found, 259.2087. 

(*R*,*E*)-Triisopropyl((3-methyl-5-(4,4,5,5-tetramethyl-1,3,2-dioxaborolan-2-yl)pent-4-en-1-yl)silane **(8).** To a solution of chromium chloride (1.37 g, 11.14 mmol) in THF (8 mL) were added sequentially a solution of (*R*)-2-methyl-4-((triisopropylsilyl)oxy)-butanal **(7)** (0.36 g, 1.39 mmol) and 2-dichloromethyl-4,4,5,5-tetramethyl-1,2,3-dioxoborolane (0.59 g, 2.28 mmol) in THF (8 mL) and a solution of LiI in THF (8 mL). The resulting mixture was stirred for 15 h at 25 °C. The mixture was poured into ice/H_2_O and the aqueous layer was extracted with Et_2_O (3x). The combined organic layers were dried with Na_2_SO_4_ and the solvent was evaporated. The residue was purified by column chromatography (silica gel, 98:2 hexane/EtOAc) to afford 0.48 g (90%) of a yellow oil identified as (*R*,*E*)-triisopropyl((3-methyl-5-(4,4,5,5-tetramethyl-1,3,2-dioxaborolan-2-yl)pent-4-en-1-yl)silane **(8)**. (α)**_D_^21^** −14° (*c* 0.56, MeOH). ^1^H-NMR (400.16 MHz, CDCl_3_): δ 6.55 (dd, *J* = 18.0, 7.2 Hz, 1H, H_4_), 5.40 (d, *J* = 18.0 Hz, 1H, H_5_), 3.68 (t, *J* = 6.7 Hz, 2H, 2H_1_), 2.47–2.34 (m, 1H, H_3_), 1.70–1.56 (m, 1H, H_2A_), 1.26 (s, 12H, –OC(CH_3_)_2_C(CH_3_)_2_O–), 1.26–1.24 (m, 1H, H_2B_), 1.06–1.04 (m, 21H, SiCH(CH_3_)_2_), 1.02 (d, *J* = 6.8 Hz, 3H, C3–CH_3_) ppm. ^13^C-NMR (101.63 MHz, CDCl_3_): δ 159.9 (d), 122.1 (d), 83.1 (q, 2x), 61.5 (t), 39.3 (d), 36.1 (t), 24.9 (2x), 24.3 (q, 2x), 19.6 (q), 18.2 (q, 6x), 12.2 (d, 3x) ppm. IR (NaCl): ν 2968 (m, C–H), 2938 (m, C–H), 2866 (m, C–H), 1636 (m), 1462 (w), 1339 (s), 1144 (m) cm^−1^. HRMS (ESI^+^): Calcd. for C_21_H_44_BO_3_Si ([M+H]^+^), 383.3144; found, 383.3151. 

**(***Z*)-3-Iodo-2-methylprop-2-en-1-ol **(10)**. CuI (0.34 g, 1.78 mmol) was added to a solution of prop-2-yn-1-ol **(9)** (1.00 g, 11.84 mmol) in Et_2_O (13.2 mL). A solution of MeMgBr (17.8 mL, 3 M in Et_2_O, 53.51 mmol) was added at −20 °C. After stirring at this temperature for 2 h, the solution was allowed to reach 25 °C and further stirred for 12 h. A solution of I_2_ (9.1 g, 35.67 mmol) in Et_2_O (40 mL) was added at 0 °C and the cooling bath was removed. After stirring at 25 °C for 24 h, the resulting mixture was cooled down to 0 °C and ice was added. The organic layer was washed with a saturated aqueous solution of Na_2_S_2_O_4_ (3x) and then filtered through a pad of Celite^®^. The residue was purified by distillation (0.2 mm Hg, 60 °C) to afford 2.1 g (60%) of a yellow oil identified as (*Z*)-3-iodo-2-methylprop-2-en-1-ol 10. ^1^H-NMR (400.16 MHz, C_6_D_6_): δ 5.49 (s, 1H, H_2_), 3.90 (s, 2H, 2H_1_), 1.55 (s, 3H, C2-CH_3_) ppm. ^13^C-NMR (101.63 MHz, C_6_D_6_): δ 146.6 (s), 74.1 (d), 67.5 (t), 20.9 (q) ppm. HRMS (ESI^+^): Calcd. for C_4_H_8_IO ([M+H]^+^), 198.9609; found, 198.9614.

(*Z*)-3-Iodo-2-methylacrylaldehyde **(11)**. To a solution of (*Z*)-3-iodo-2-methylprop-2-en-1-ol **(10)** (0.21 g, 1.06 mmol) in CH_2_Cl_2_ (53 mL) were sequentially added MnO_2_ (0.93 g, 10.65 mmol) and Na_2_CO_3_ (1.13 g, 10.65 mmol) and the reaction mixture was stirred for 1 h at 25 °C. Then, the mixture was filtered through a pad of Celite^®^ washing with CH_2_Cl_2_. The solvent was evaporated to afford 0.15 g (73%) of a yellow oil identified as (*Z*)-3-iodo-2-methylacrylaldehyde **(11)**. The spectroscopic data are identical to those previously reported [21]. 

2-(1*E*,3*Z*)-4-Iodo-3-methylbuta-1,3-dien-1-yl)-1,3,3-trimethylcyclohex-1-ene **(13**). To a cold (−30 °C) solution of phosphonium salt **(12)** [22] (0.15 g, 0.31 mmol) in THF (2.5 mL), *n*BuLi (0.9 mL, 2.36 M in hexanes, 0.36 mmol) was added and the mixture was stirred at 0 °C for 45 min. After cooling down to −30 °C, a solution of (*Z*)-3-iodo-2-methylacrylaldehyde **(11)** (0.07 g, 0.36 mmol) in THF (2.5 mL) was added and the reaction mixture was stirred for 1.5 h. Water (5 mL) was added and the mixture was extracted with hexane (3x). The organic extracts were dried (Na_2_SO_4_) and the solvent was evaporated. The residue was purified by column chromatography (C18-silica gel, CH_3_CN) to afford 65 mg (64%) of a yellow oil identified as 2-(1*E*,3*Z*)-4-iodo-3-methylbuta-1,3-dien-1-yl)-1,3,3-trimethylcyclohex-1-ene **(13)**. ^1^H-NMR (400.16 MHz, C_6_D_6_): δ 6.80 (d, *J* = 16.1 Hz, 1H), 6.32 (d, *J* = 16.1 Hz, 1H), 5.80 (s, 1H), 1.90–1.86 (m, 2H), 1.78 (s, 3H, CH_3_), 1.66 (s, 3H, CH_3_), 1.57–1.48 (m, 2H), 1.44–1.39 (m, 2H), 1.07 (s, 6H, 2xCH_3_) ppm. ^13^C-NMR (101.63 MHz, C_6_D_6_): δ 142.3 (s), 137.5 (s), 135.1 (d), 132.1 (d), 130.8 (s), 78.6 (d), 39.7 (t), 34.0 (s), 33.1 (t), 29.1 (q, 2x), 22.0 (q), 20.9 (q), 19.3 (t) ppm. UV (MeOH): λ_max_ 266 nm. IR (NaCl): ν 2925 (s, C–H), 2861 (m, C–H), 1441 (m), 741 (s) cm^−1^. HRMS (ESI^+^): Calcd. for C_14_H_21_I ([M+H]^+^), 316.0688; found, 316.0684.

Silyl Ether **(14)**. To a solution of 2-(1*E*,3*Z*)-4-iodo-3-methylbuta-1,3-dien-1-yl)-1,3,3-trimethylcyclohex-1-ene **(13)** (0.16 g, 0.51 mmol) in THF (0.2 mL) was added Pd(PPh_3_)_4_ (45 mg, 0.039 mmol). After stirring for 5 min at 25 °C, a solution of (*R*)-alkenylborane **(8)** (150 mg, 0.392 mmol) in THF (1.0 mL) and a 10% aqueous solution of TlOH (4.26 mL) were added and the reaction mixture was stirred for 2 h. The mixture was extracted with Et_2_O (3x) and the combined organic layers were washed with brine (3x) and dried (Na_2_SO_4_) and the solvent was evaporated. The residue was purified by column chromatography (silica gel, 95:5 hexane/EtOAc) to afford 0.12 g (71%) of a colourless oil identified as silyl ether **(14)**. (α)**_D_^20^** 15.8° (*c* 0.25, MeOH). ^1^H-NMR (400.16 MHz, C_6_D_6_): δ 6.94 (d, *J* = 16.0 Hz, 1H, H_7_), 6.76 (dd, *J* = 14.9, 11.1 Hz, 1H, H_11_), 6.27 (d, *J* = 16.0 Hz, 1H, H_8_), 6.04 (d, *J* = 11.1 Hz, 1H, H_10_), 5.52 (dd, *J* = 14.9, 8.3 Hz, 1H, H_12_), 3.69–3.65 (m, 2H, 2H_15_), 2.55–2.46 (m, 1H, H_13_), 1.99–1.93 (m, 2H, 2H_4_), 1.92 (s, 3H, CH_3_), 1.81 (s, 3H, CH_3_), 1.64–1.57 (m, 2H, 2H_3_), 1.55–1.51 (m, 1H, H_14A_), 1.52–1.45 (m, 2H, 2H_2_), 1.14–1.11 (m, 21H, 3xSi*^i^*Pr_3_), 1.11 (s, 6H, 2xCH_3_), 1.08–1.02 (m, 1H. H_14B_), 1.00 (d, *J* = 6.8 Hz, 3H, CH_3_) ppm. ^13^C-NMR (101.63 MHz, C_6_D_6_): δ 139.9 (d), 138.6 (s), 132.4 (s), 131.0 (d), 129.6 (d), 129.1 (s), 127.9 (s), 125.2 (s), 61.7 (t), 40.6 (t), 39.9 (t), 34.5 (s), 34.2 (d), 33.3 (t), 29.2 (q), 22.0 (q), 21.2 (q), 20.7 (q), 19.8 (t), 18.4 (q, 6x), 12.4 (d, 3x) ppm. IR (NaCl): ν 2971 (s, C–H), 2864 (s, C–H), 1462 (m, C–H), 1365 (m) 1105 (s), 965 (s) cm^−1^. HRMS (ESI^+^): Calcd. for C_29_H_53_OSi ([M+H]^+^), 445.3846; found, 445.3860.

(*R*)-9-*cis*-13,14-Dihydroretinol (**(2)**; 9CDHROL). To a cooled (0 °C) solution of silyl ether **(14)** (91.2 mg, 0.212 mmol) in THF (3.5 mL) was added *n*Bu_4_NF (0.32 mL, 1M in THF, 0.32 mmol) and the mixture was stirred for 0.5 h at 25 °C. A saturated aqueous solution of NaHCO_3_ was added and the mixture was extracted with Et_2_O (3x). The combined organic layers were washed with brine (3x) and dried (Na_2_SO_4_) and the solvent was evaporated. The residue was purified by column chromatography (gradient from 95:5 to 70:30 hexane/EtOAc) to afford 41 mg (67%) of a colorless oil identified as (*R*)-9-*cis*-13,14-dihydroretinol **(2)**. (α)**_D_^21^** -9.4° (*c* 0.37, MeOH). ^1^H-NMR (400.16 MHz, C_6_D_6_): δ 6.93 (d, *J* = 16.0 Hz, 1H), 6.69 (dd, *J* = 15, 11.1 Hz, 1H), 6.29 (d, *J* = 16.0 Hz, 1H), 6.01 (d, *J* = 11.1 Hz, 1H), 5.46 (dd, *J* = 15.0, 8.3 Hz, 1H), 3.35 (t, *J* = 6.6 Hz, 2H), 2.34–2.19 (m, 1H), 1.99–1.89 (m, 2H), 1.93 (s, 3H, CH_3_), 1.79 (s, 3H, CH_3_), 1.62–1.53 (m, 2H), 1.50–1.44 (m, 2H), 1.36 (q, *J* = 6.7 Hz, 2H), 1.11 (s, 6H, 2xCH_3_), 0.91 (d, *J* = 6.7 Hz, 3H, CH_3_) ppm. ^13^C-NMR (101.63 MHz, C_6_D_6_): δ 140.0 (d), 138.6 (s), 132.5 (s), 130.9 (d), 129.6 (d), 129.2 (s), 128.0 (d), 124.9 (d), 60.9 (t), 40.2 (t), 39.9 (t), 34.5 (s), 34.3 (d), 33.2 (t), 29.21 (q), 29.19 (q), 22.0 (q), 20.9 (q), 20.7 (q), 19.7 (t) ppm. IR (NaCl): ν 3500–3300 (br, O-H), 2926 (s, C–H), 1451 (m, C–H), 965 (s) cm^−1^. HRMS (ESI^+^): Calcd. for C_20_H_33_O ((M+H)^+^), 289.2584; found, 289.2525.

(*R*)-9-*cis*-13,14-Dihydroretinal **(15)**. To a solution of (*R*)-9-*cis*-13,14-dihydroretinol **(2)** (25 mg, 0.087 mmol) in CH_2_Cl_2_ (3.7 mL) at 0 °C were added Dess-Martin periodinane (73.5 mg, 0.173 mmol) and pyridine (0.014 mL, 0.173 mmol) and the reaction mixture was stirred for 30 min at 0 °C and for 1 h at 25 °C. Then, Et_2_O (5 mL), a saturated aqueous solution of NaHCO_3_ (3 mL) and a saturated aqueous solution of Na_2_S_2_O_3_ (3 mL) were added. The layers were separated, the aqueous layer was extracted with Et_2_O (3x), the combined organic layers were washed with a saturated aqueous solution of NaHCO_3_ (2x) and brine (2x), dried (Na_2_SO_4_) and the solvent was evaporated. The residue was purified by column chromatography (silica gel, gradient from 95:5 to 70:30 hexane/EtOAc) to afford 14 mg (57%) of a colorless oil identified as (*R*)-9-*cis*-13,14-dihydroretinal **(15)**. (α)**_D_^21^** 1.5° (*c* 0.30, MeOH). ^1^H-NMR (400.16 MHz, C_6_D_6_): δ 9.27 (t, *J* = 2.0 Hz, 1H, CHO), 6.91 (d, *J* = 16.0 Hz, 1H, H_7_), 6.60 (dd, *J* = 14.8, 11.0 Hz, 1H, H_11_), 6.30 (d, *J* = 16.0 Hz, 1H, H_8_), 5.93 (d, *J* = 11.0 Hz, 1H, H_10_), 5.36 (dd, *J* = 15.1, 7.5 Hz, 1H, H_12_), 2.53–2.43 (m, 1H, H_13_), 1.98–1.93 (m, 2H_3_), 1.91 (s, 3H, CH_3_), 1.88 (dd, *J* = 6.7, 1.9 Hz, 1H, H_14A_), 1.81 (s, 3H, CH_3_), 1.81–1.77 (d, *J* = 2.1 Hz, 1H, H_14B_), 1.63–1.53 (m, 2H, 2H_2_), 1.50–1.44 (m, 2H, 2H_4_), 1.38 (s, 3H, CH_3_), 1.12 (s, 6H, 2xCH_3_) ppm. ^13^C-NMR (101.63 MHz, C_6_D_6_): δ 200.1 (s), 138.6 (s), 137.7 (d), 133.2 (s), 130.8 (d), 129.4 (d), 129.1 (s), 128.7 (d), 125.1 (d), 50.4 (t), 39.9 (t), 34.5 (s), 33.3 (q), 31.9 (d), 30.3 (t), 29.2 (t), 22.1 (q), 20.7 (q), 20.4 (q), 19.7 (t), 14.4 (q) ppm. IR (NaCl): ν 2920 (s, C–H), 2854 (s, C–H), 1458 (w), 965 (w) cm^−1^. HRMS (ESI^+^): Calcd. for C_20_H_31_O ([M+H]^+^), 287.2362; found, 287.2369.

(2*E*,4*E*,6*E*,8*E*)-(3,7-Dimethyl-9-(2,6,6-trimethylcyclohex-1-en-1-yl)nona-2,4,6,8-tetraen-1-yl)triphenyl-phosphonium Chloride **(17)**. To a solution of all-*trans*-retinol **(16)** (1.37 g, 4.78 mmol) in MeOH (2.7 mL) was added PPh_3_ (1.44 g, 5.5 mmol) and a 4 M solution of HCl in dioxane (1.4 mL) and the reaction mixture was stirred for 2 h at 25 °C. Then, the mixture was poured into water and extracted with Et_2_O (2x). The aqueous layer was extracted with EtOAc (3x), the combined organic layers were dried (Na_2_SO_4_) and the solvent was evaporated. The light orange residue was triturated three times with EtOAc to afford 1.39 g (52%) of a yellow solid identified as (2*E*,4*E*,6*E*,8*E*)-(3,7-dimethyl-9-(2,6,6-trimethylcyclohex-1-en-1-yl)nona-2,4,6,8-tetraen-1-yl)triphenyl-phosphonium chloride **(17)**. ^1^H-NMR (400.16 MHz, CDCl_3_): δ 7.94–7.83 (m, 6H), 7.82–7.74 (m, 3H), 7.73–7.63 (m, 6H), 6.49 (m, 1H), 6.26–5.88 (m, 4H), 5.39 (m, 1H), 5.09–4.97 (m, 2H), 2.01 (d, *J* = 6.1 Hz, 2H, CH_2_), 1.91 (s, 3H, CH_3_), 1.69 (s, 3H, CH_3_), 1.63 (s, 6H, 2xCH_3_), 1.48 (m, 4H, 2xCH_2_), 1.01 (s, 3H, CH_3_) ppm. ^13^C-NMR (101.63 MHz, CDCl_3_): δ 143.8 (s, *J*_C-P_ = 2.9 Hz, 3x), 137.7 (s), 137.5 (s), 135.0 (d, *J*_C-P_ = 2.9 Hz, 3x), 133.9 (d, *J*_C-P_ = 9.7 Hz, 6x), 132.0 (d, *J*_-P_ = 12.4 Hz), 130.3 (d, *J*_C-P_ = 12.4 Hz, 6x), 129.5 (d, *J*_C-P_ = 2.9 Hz), 128.5 (d, *J*_C-P_ = 12.2 Hz), 127.5 (d), 126.4 (d, *J*_C-P_ = 5.2 Hz), 118.7 (s), 117.8 (s), 114.2 (d, *J*_C-P_ = 12.1 Hz), 39.6 (t), 34.2 (s), 33.0 (t), 28.9 (q, 2x), 25.4 (t), 24.9 (t), 19.2 (q), 13.1 (q), 12.8 (q) ppm. HRMS (ESI^+^): Calcd. for C_38_H_44_P ([M-Cl]^+^), 531.3175; found, 531.3166.

(*R*)-9-*cis*-13,14-Dihydro-β,β-carotene (**(18)**; 9CDHBC). To a cooled (−78 °C) solution of phosphonium chloride **(17)** (24.9 mg, 0.044 mmol) in THF (0.2 mL) was added *n*BuLi (0.027 mL, 1.6 M in hexanes, 0.044 mmol) and the reaction mixture was stirred for 30 min. Then a solution of (*R*)-9-*cis*-13,14-dihydroretinal **(15)** (9.0 mg, 0.031 mmol) in THF (0.13 mL) was added and the mixture was stirred for 1 h at −78 °C and for 30 min at 25 °C. Water was added and the mixture was extracted with Et_2_O (3x). The combined organic layers were washed with a saturated aqueous solution of NaCl (2x) and dried (Na_2_SO_4_). The solvent was evaporated and the residue was purified by column chromatography (C18-silica gel, CH_3_CN) to afford 11.3 mg (67%) of a red foam identified as (*R*)-9-*cis*-13,14-dihydro-β,β-carotene **(18)**. It was observed that this product was very unstable and rapidly degraded. ^1^H-NMR (400.16 MHz, acetone-d_6_): δ 6.69–6.60 (m, 2H), 6.56 (dd, *J* = 14.4, 11.2 Hz, 1H, H_11′_), 6.48 (dd, *J* = 14.9, 11.1 Hz, 1H, H_15_), 6.34 (d, *J* = 15.1 Hz, 1H), 6.21–6.13 (m, 4H), 6.12 (dd, *J* = 11.5, 7.8 Hz, 1H), 5.94 (d, *J* = 11.7 Hz, 1H, H_10′_), 5.81–5.71 (m, 1H, H_15′_), 5.62 (dd, *J* = 15.0, 8.2 Hz, 1H, H_12′_), 2.44–2.33 (m, 1H, H_13′_), 2.25–2.19 (m, 2H, 2H_14′_), 2.05–2.00 (m, 4H), 1.96 (s, 3H, CH_3_), 1.92 (s, 3H, CH_3_), 1.91 (s, 3H, CH_3_), 1.72 (s, 3H, CH_3_), 1.70 (s, 3H, CH_3_), 1.67–1.56 (m, 4H), 1.50–1.45 (m, 4H), 1.04 (s, 3H, CH_3_), 1.03 (s, 12H, 4xCH_3_) ppm. ^13^C-NMR (101.63 MHz, acetone-d_6_): δ 140.3 (d), 139.1 (d), 139.0 (s), 138.9 (s), 138.7 (d), 134.9 (d), 134.8 (s), 133.1 (s), 132.8 (d), 132.2 (d), 131.3 (d), 130.0 (d, 2x), 129.8 (s), 129.7 (s), 129.6 (d), 128.5 (d), 127.1 (d), 126.2 (s), 125.4 (d), 41.6 (t, 2x), 40.6 (t), 40.7 (t), 38.3 (d), 35.1 (s), 35.0 (s), 30.9 (t, 2x), 30.8 (q), 29.5 (q, 4x), 22.2 (q), 22.1 (q), 20.8 (q), 20.7 (q), 20.1 (t), 12.9 (q) ppm. UV (MeOH): λ_max_ 324 nm. HRMS (ESI^+^): Calcd. for C_40_H_59_ ((M+H)^+^), 539.4604; found, 539.4611.

#### 2.1.3. *Synthesis of 9-cis-β,β-Carotene (9CBC; Figure 1C, including Numbering)*

Ethyl 9-*cis*-Retinoate **(21)**. To a cooled (0 °C) solution of ethyl (E)-4-(diethoxyphosphoryl)-3-methylbut-2-enoate **(20)** (0.227 g, 1.10 mmol) in THF (2.0 mL) was added nBuLi (0.63 mL, 1.00 mmol, 1.6 M in hexane) and DMPU (0.15 mL, 1.24 mmol). After stirring for 1 h, the reaction was cooled down to −78 °C and a solution of (2*Z*,4*E*)-3-methyl-5-(2,6,6-trimethylcyclohex-1-en-1-yl)penta-2,4-dienal **(19)** (0.1 g, 0.46 mmol) in THF (2.5 mL) was added and the mixture was stirred for 2 h. Water was added and the mixture was extracted with Et_2_O (3x). The combined organic layers were dried (Na_2_SO_4_) and the solvent was evaporated. The residue was purified by column chromatography (silica gel, 95:5 hexane/EtOAc) to afford 0.144 g (96%) of a yellow solid identified as ethyl 9-*cis*-retinoate **(21)**. The spectroscopic data are identical to those previously reported [22].

9-*cis*-Retinol **(22)**. To a cooled (−78 °C) solution of ethyl 9-*cis*-retinoate **(21)** (0.154 g, 0.47 mmol) in THF (2.3 mL), DIBAL-H (1.08 mL, 1.08 mmol, 1.0 M in toluene) was added and the reaction was stirred for 2 h. Water was added and the mixture was extracted with EtOAc (3x). The combined organic layers were dried (Na_2_SO_4_) and the solvent was evaporated. The residue was purified by column chromatography (silica gel, from 95:5 to 80:20 hexane/EtOAc) to afford 0.08 g (60%) of a yellow oil identified as 9-*cis*-retinol **(22).** The spectroscopic data are identical to those previously reported [23].

9-*cis*-Retinal **(23)**. To a solution of 9-*cis*-retinol **(22)** (65 mg, 0.23 mmol) in CH_2_Cl_2_ (9.1 mL) were added MnO_2_ (197 mg, 2.27 mmol) and Na_2_CO_3_ (240 mg, 2.27 mmol) and the reaction mixture was stirred for 1.5 h at 25 °C. The mixture was filtered through a pad of Celite^®^ washing with CH_2_Cl_2_. The solvent was evaporated to afford 38 mg (58%) of a yellow oil identified as 9-*cis*-retinal **(23)**. The spectroscopic data matched those previously reported [24].

9-*cis*-β,β-Carotene (**(24)**, 9CBC). To a solution of phosphonium salt **(17)** (24.9 mg, 0.044 mmol) in THF (0.20 mL) at −78 °C was added *n*BuLi (0.027 mL, 1.6 M in hexane, 0.044 mmol) and the resulting mixture was stirred for 30 min. Then, a solution of 9-*cis*-retinal **(23)** (9.0 mg, 0.031 mmol) in THF (0.13 mL) was added and the mixture was stirred for 1 h at −78 °C and for 30 min at 25 °C. Water was added and the mixture was extracted with Et_2_O (3x). The combined organic layers were washed with brine (2x) and dried (Na_2_SO_4_) and the solvent was evaporated. The residue was purified by column chromatography (C18-silica gel, CH_3_CN) to afford 11.3 mg (67%) of a red foam identified as 9-*cis*-β,β-carotene **(24)**. ^1^H-NMR (400.16 MHz, CDCl_3_): δ 6.74 (dd, *J* = 14.8, 11.6 Hz, 2H), 6.68 (d, *J* = 7.9 Hz, 1H), 6.66–6.54 (m, 3H), 6.28 (d, *J* = 14.9 Hz, 1H), 6.26–6.19 (m, 2H), 6.19–6.08 (m, 4H), 6.05 (d, *J* = 11.5 Hz, 1H), 2.08–1.99 (m, 4H), 1.97 (s, 3H), 1.95 (s, 6H), 1.76 (s, 3H), 1.71 (s, 3H), 1.66–1.59 (m, 4H), 1.51–1.39 (m, 4H), 1.25 (s, 3H), 1.04 (s, 6H), 1.03 (s, 6H) ppm. ^13^C-NMR (101.63 MHz, CDCl_3_): δ 138.4 (s), 138.1 (s), 137.8 (d), 136.7 (d), 136.4 (s, 2x), 136.1 (s), 134.6 (d), 130.9 (d), 132.4 (d), 130.2 (d, 3x), 130.0 (d), 129.6 (s, 2x), 129.5 (d, 2x), 128.5 (d), 127.1 (s), 127.0 (d), 123.8 (d), 39.7 (t, 2x), 34.4 (s, 2x), 33.3 (t, 2x), 29.1 (q, 2x), 28.6 (q, 2x), 22.0 (q), 21.9 (q), 20.9 (q), 19.4 (t, 2x), 13.0 (q), 12.9 (q) ppm. UV (MeOH): λ_max_ 397 nm. HRMS (ESI^+^): Calcd. for C_40_H_56_ ([M]^+^), 536.4375; found, 536.4376. The spectroscopic data matched those previously reported [25,26].

### 2.2. Samples Used for LC-MS Analysis

Food samples: Beef liver (n = 3) was bought at a local butcher in Vigo, Spain. Canned peaches (n = 3; Metades, Pessago em calda, Auchan/Alcampo-home-brand/420 g can) were purchased at Alcampo, Vigo, Spain. 

Human serum samples (n = 3) were obtained from the blood of healthy volunteers with the subjects’ written informed consent.

### 2.3. Animal Experiments

#### 2.3.1. *Animals*

Wild-type (WT) C57BL6N male mice (Charles River, France) used for metabolic analyses were housed in groups of 4–5 mice per cage in a 7 a.m.–7 p.m. light/dark cycle in individually ventilated cages (Techniplast, Italy). RXRγ-/- and control WT mice were raised on mixed C57BL6N and 129SVpas genetic background as described [27]. Food (standard chow diet, D04 from SAFE, France) and water were freely available. All animal care and experimentation were carried out in accordance with European Union Council (2010/63/EU) and the French Ministry of Agriculture (87848) guidelines for the use of laboratory animals in behavioural studies. The French National Ethics Committees approved all experimental protocols under specific authorization (No. 2016022411354542). Accordingly, animal experimentation and statistical analyses were planned to minimize the number of animals used, within the constraints of necessary power.

#### 2.3.2. *Animal Treatments*

ATROL, 9CDHROL and 9-*cis*-13,14-dihydro-β,β-carotene (9CDHBC) were dissolved in ethanol, and then mixed with sunflower oil, so that the final solution contained 3% ethanol. Vehicle treatments consisted of 3% ethanol solution in sunflower oil. Treatments were administered *per os* as a single dose at 40 mg/kg for each substance and volume/weight ratio 3 mL/kg for chemical analyses. Mice were always treated during the active dark phase of the Light/Dark cycle at 10 pm and samples were collected 11 h later in the light protected conditions, weighted, frozen in liquid nitrogen and stored at −80 °C until analyses. Serum was prepared and immediately stored in brown vials at −80 °C until further analysis. For memory tests (see below) n = 8 for WT and n = 5 for RXRγ-/- mice were injected with a single dose of 40 mg/kg of 9CDHROL at volume/weight ratio of 3 mL/kg and 9–12 h before testing. Such a long latency was selected to allow not only the most complete metabolic conversion of 9CDHROL to 9CDHRA, but also induction of transcriptional programs controlled by RXRs including translation and maturation of transcribed proteins prior to testing. To establish whether vehicle treatment might affect animal performance we analyzed memory in mice treated with vehicle alone. This control testing session took place 48 h before the testing session with 9CDHROL treatment.

#### 2.3.3. *Memory Analysis*

Working memory analysis was carried out in the Institute Clinique de la Souris (http://www.ics-mci.fr/ accessed on 7 March 2020) according to standard operating procedures. 12-week old male mice (*n* = 10 WT and *n* = 8 RXRγ-/-) were trained in the DNMTP in the T-maze using 5 trials per day according to a protocol previously described [15] with modifications to facilitate pharmacological tests [27]. Briefly, all mice were first trained over 10 days to acquire procedural memory in delayed non-match to place (DNMTP) paradigm in the T-maze. Each day, mice ran for a reward of 25% sucrose during 5 trials. Each trial was composed of a learning phase (only one arm was opened and baited) and a testing phase (two arms opened, but only the newly opened arm was baited) separated by a minimal (15 s) inter-trial interval (ITI). The percentage of correct choices (choice of rewarded arm) was calculated for each animal every day. Minimum 4 correct choices out of total of 5 choices (80%) over 3 consecutive days was considered as criterion of acquisition and mice which did not acquire this criterion were excluded from the study (*n* = 2 WT and *n* = 3 RXRγ-/-). Thus, final number of mice taken into account for analyses was *n* = 8 for WT and *n* = 5 for RXRγ-/- group. Since the results were significant and consistent with previous observations [27], the cohort numbers were not increased, reducing thus the number of animals used. After a training period of 10 days the ITIs were extended from 3 to 24 min in order to establish the minimum time at which mouse performed at chance (60% of correct choices or less). These ITIs were next used for testing promnemonic activities of treatments. 

### 2.4. Cell Cultures and Pharmacological Treatments

158N mouse oligodendrocyte cells [28] (kindly provided by Dr M. Said Ghandour, Virginia Commonwealth University, USA) were maintained in culture in DMEM supplemented with 5% calf serum (Invitrogen, France). After reaching confluency of 80% cells were treated with 10^−3^ M ethanol solutions of 9CDHROL, 9CDHROL-ES (as an acetate-ester), 9CDHRA, 9-*cis*-dihydroretinoyl-ester (9CDHRA-ES, as an ethyl-ester) or ATROL, or 10^−3^ M DMSO solutions of 9CDHBC, 9CBC and ATBC to attain final concentration of 10^−6^ M for each compound. Ethanol at corresponding concentration was used as control vehicle treatment. Each treatment was performed in triplicate providing thus *n* = 3 independent biological samples, each sample obtained by pulling cells from 3 independent flasks to produce a cell pellet of about 120–150 mg and corresponding to about 60 million cells. Briefly, cells were harvested 18 h after treatment using isotonic, non-enzymatic cell dissociation buffer (Invitrogen, ref. 13150-016). After centrifugation and weighting, all samples were frozen in liquid nitrogen and stored at −80° until analyses. All procedures were carried out in light protected conditions to avoid photochemical degradation of retinoids.

### 2.5. Liquid Chromatography-Mass Spectrometry (LC-MS) Analysis—Combined Retinoid and Carotenoid Analysis

Analytical procedures: Analyses were performed under dark yellow/amber light using previously sample preparation methodology as a validated protocol [29] for RA-isomer and ROL analysis. The basic methodology [29] was improved using a more sensitive MS setup using a high performance liquid chromatography mass spectrometry (Agilent 1260 Infinity LC system; Madrid, Spain)–mass spectrometry (SCIEX Triple Quad 3500 System; Sciex, Madrid, Spain), plus and additional online diode array detector (Waters 966 DAD, Waters, Santiago de Compostela, Spain) system. In addition to the first and second eluents already mentioned in [29] a third and fourth eluent after 20 min elution time were used. Linear gradient from 20 min 20% (isopropanol:methanol:methyl-tert-butyl-ether (MTBE)/30:30:40)—80% (isopropanol:methanol/50:50), 25 min 40% (isopropanol:methanol:MTBE/30:30:40)—60% (isopropanol:methanol/50:50), 29 min 70% (isopropanol:methanol:MTBE/30:30:40)—30% (isopropanol:methanol/50:50), 30 min 0% (isopropanol:methanol:MTBE/30:30:40)—100% (isopropanol:methanol/50:50), 30.1 min 20% (isopropanol:methanol:MTBE/30:30:40)—80% (isopropanol:methanol/50:50). For the detection of retinoic acids MS-MS settings 301 -> 205 *m*/*z*, 13,14-dihydroretinoic acid MS-MS setting 303 -> 207 *m*/*z*, for the detection of 13,14-dihydroretinol MS-MS settings 290 -> 69 *m*/*z* and for the detection of 9CDHBC 405 -> 405/405 -> 95 *m*/*z* were used while for 9CDHBC additionally a diode array detector at 366 nm and for 9CBC and ATBC exclusively a diode array detector at 411 nm were used. For each additional analyzed substance (ATDHRA, 9CDHRA, ATDHROL, 9CDHROL, ATBC, 9CBC and 9CDHBC), a linearization, recovery determination, *intra*- and *inter*-day variability and limit of quantification and limit of determination was performed and implemented in the concentration calculation process. The standard compounds used were synthesized as described in the manuscript earlier, obtained as described in [29], or alternatively for 9CDHRA and ATDHRA as previously published [11]. Accordingly, for sample preparation 100 mg of the material (if samples were under 100 mg, water was added up to the used standard weight: 100 mg) or 100 μL serum was diluted with a threefold volume of isopropanol. The tissues were minced by scissors, vortexed for 10 s, put in an ultrasonic bath for 5 min, shaken for 6 min and centrifuged at 13,000 rpm in a Heraeus BIOFUGE Fresco at +4 °C. After centrifugation, the supernatants were dried in a GYROZEN centrifugal vacuum concentrator equipped with an ILMAC MPC 301-Z vacuum pump (CONTROLTECNICA, Madrid, Spain) at 30 °C. The dried extracts were resuspended with 30 μL of methanol—MTBE (50:50) and transferred into the auto sampler and 10 µL subsequently analyzed.

### 2.6. Statistical Analysis

Statistical analyses for behavioral data (shown in Supplementary Figure S1) were performed using two-way ANOVA, or for procedural learning two-way ANOVA, with repeated measures and followed by *post-hoc* Bonferroni test. Analyses of retinoid/carotenoid metabolism as well as ITI analyses in DNMTP behavioral test were performed using student t-test versus control/WT with a statistically significance accepted at *p* < 0.05. Significant differences are indicated in the corresponding figures and figure legends.

## 3. Results

### 3.1. Identification of Endogenous 9CDHROL in Mouse, Human and the Human Food Matrix

**Figure 2:**
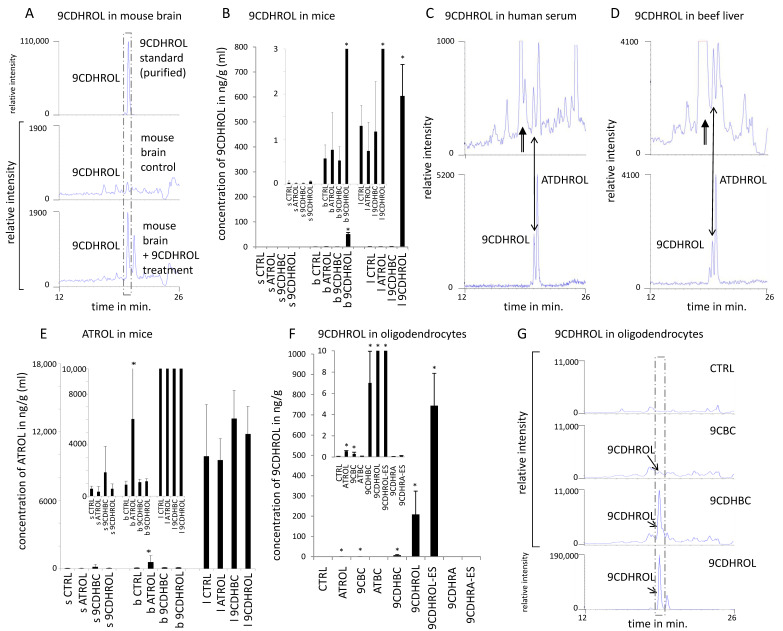
**9CDHROL as a novel endogenous and nutritionally-relevant retinoid**. (**A**). 9CDHROL (indicated by a dashed line box) standard (top chromatogram) and endogenous levels in mouse brain (middle chromatogram), and after 9CDHROL-treatment (bottom chromatogram) (§, §§) ($). (**B**). 9CDHROL levels in mouse organs/compartments (each n = 5, s—serum, b—brain, l—liver) after single *per os* treatment with vehicle for control group (CTRL), or 40 mg/kg of ATROL, 9CDHBC or 9CDHROL (€). (**C**). 9CDHROL in human serum (top chromatogram) and 9CDHROL standard mixture including ATDHROL (all-*trans*-dihydroretinol) (bottom chromatogram) (§§). Double lined arrows indicate unknown peaks and the double sided arrow indicate 9CDHROL in comparison to the standard ($). (**D**). 9CDHROL in human food chain/beef liver (top chromatogram) and 9CDHROL standard mixture including ATDHROL (bottom chromatogram) ($). The double lined arrows indicate unknown peaks and the double sided arrow indicate 9CDHROL in comparison to the standard. (**E**). Concentrations of ATROL in serum (s), brain (b) and liver (l), each *n* = 5. ATROL levels before and after retinol treatment in serum, brain and liver (€). (**F**). 9CDHROL levels including standard deviation in cultured mouse oligodendrocytes after control treatment using ethanol (CTRL) or ATROL-, 9CBC-, ATBC-, 9CDHBC-, 9CDHROL-, 9CDHROL-ES-, 9CDHRA- or 9CDHRA-ES-treatment, each at 10^−6^ M and *n* = 3 (€). (**G**). Conversion of 9CDHBC and 9CBC to 9CDHROL (indicated by a dashed line box) in mouse oligodendrocyte cell line *in vitro* after ethanol (CTRL) or 9CBC, 9CDHBC and 9CDHROL administration (§, §§).

By analogy to ATROL acting as the main precursor of ATRA, we investigated whether 9CDHROL could be a physiological precursor of 9CDHRA. To address this hypothesis, we first obtained by targeted chemical synthesis high purity 9CDHROL (Figure 1A). Using it as a standard in our LC-MS analytics (Figure 2A, top panel) we showed that 9CDHROL is present endogenously in mice as illustrated for brain (0.6 ng/g, Figure 2A) and additionally quantified for serum and liver (Figure 2B, control levels). Importantly, based on the co-elution and using the same LC-MS parameters and the same fragmentation pattern channels (290 -> 69 *m*/*z*), we also determined the presence of 9CDHROL in human serum (0.9 ng/mL; Figure 2C) and beef liver, as an example of human food matrix (8 ng/g; Figure 2D). Thus, we have identified 9CDHROL as a new, endogenous retinoid present in mice, humans and in the human food chain. Further parallel analytical proof of endogenous occurring 9CDHROL could not be performed because of too low endogenous levels.

(§)—Y-axis scales, displaying relative intensity in 290 -> 69 m/z were (partly) identical; (§§)—Y-axis scales (relative intensity in 290 -> 69 m/z) were (partly) fit to the maximum height of the relevant peaks; (€)—inserted is an additional Figure with a specifically set y-axis for improved visualization of relevant lower values; (*)—statistical significance in (B,E,F) is marked with a star over the bar, when p < 0.05; ($)—after getting the best sensitivity on the MS-MS detection mode employed at 290 -> 69 m/z, the presence of additional co-eluting peaks with similar fragmentation pattern like various dihydroretinol-derivatives/acyclo-dihydroretinol-derivatives with hydrogenated double bounds at various positions and their geometric isomers could be determined. We are currently in the process of carefully identifying these derivatives.

Endogenous levels of 9CDHROL can likely be increased by diet as its levels in the liver rose by ca. 600-fold 11 h after oral 9CDHROL treatment in mice (40 mg/kg bw). Such increase of 9CDHROL after 9CDHROL treatment was also significant, but less pronounced in the brain (ca. 90-fold increase, Figure 2A,B) and not observable in the serum, pointing to tissue specific biodistribution and/or accumulation. ATROL levels following treatment with equivalent amounts of ATROL increased just in the brain (Figure 2E), suggesting an important function and requirement of vitamin A1 in the brain [30,31]. Serum and liver ATROL levels were not significantly altered 11 hrs after treatment, supporting a dynamic homeostatic regulation in the blood and liver (Figure 2E) [32,33]. It was striking, however, that despite single treatment with high dose of 9CDHROL the liver levels of 9CDHROL were ca. 15-times lower than basal ATROL levels (compare Figure 2B,E). In contrast, 9CDHROL levels in the brain after 9CDHROL treatment easily reach an equivalent range of ATROL measured after ATROL treatment (compared to our own data shown in Figure 2B,E addressing the control-levels of ATROL), pointing towards an important function and selective requirement of 9CDHROL and further RXR-mediated signaling in the brain. Importantly, we did not detect any increase of 9CDHROL levels after ATROL treatment (Figure 2B) or inversely an increase of ATROL levels after 9CDHROL treatment (Figure 2E) in the plasma, liver or brain, indicating that direct interconversion of these two retinoid structures is not possible or is highly limited in physiological conditions *in vivo*. However, some conversion or biological feedback may appear in specific cell types, which is supported by weak, but significant increase of 9CDHROL levels after *in vitro* treatment with ATROL of 158N oligodendrocytes (Figure 2F,G). 

### 3.2. The Vitamin A5/X Concept: 9CDHROL, but Not ATROL, Is an Efficient Precursor of 9CDHRA in Mice

**Figure 3:**
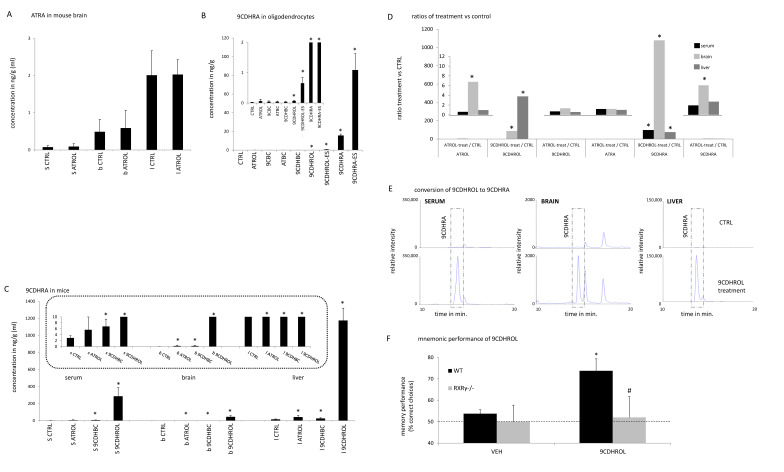
**Endogenous and nutritionally-relevant precursors of 9CDHRA and specific RXR-mediated signaling properties of 9CDHROL**. (**A**). Concentrations of ATRA in serum (s), brain (b) and liver (l). All-*trans*-retinoic acid (ATRA) levels before and after retinol treatment in serum, brain and liver, each *n* = 5. (**B**). 9CDHRA levels including standard deviation in cultured mouse oligodendrocytes after treatment with ethanol (CTRL), or ATROL-, 9CBC-, ATBC-, 9CDHBC-, 9CDHROL-, 9CDHROL-ES-, 9CDHRA and 9CDHRA-ES, each at 10^−6^ M and *n* = 3 (€). (**C**). 9CDHRA levels including standard deviation in mouse organs (each *n* = 5, s—serum, b—brain, l—liver) after control treatment (CTRL), ATROL-, 9CDHBC- or 9CDHROL-treament each with an oral gavage of 40 mg/kg (€). (**D**). Concentrations of lipid hormones and nuclear hormone receptor activating ligands (ATRA and 9CDHRA) as well as their nutritional precursors (ATROL and 9CDHROL) in serum (s), brain (b) and liver (l) summarized for 9CDHROL and ATROL. Summarized ratios of substance treatment (treat) vs. CTRL treatment (CTRL), specifically for “ATROL”: ATROL-treat/CTRL, “9CDHROL”: 9CDHROL-treat/CTRL, or ATROL-treat/CTRL and for “ATRA”: ATROL-treat/CTRL and for “9CDHRA”: 9CDHROL-treat/CTRL or ATROL-treat/CTRL (€). (**E**). Conversion of 9CDHROL to 9CDHRA, 9CDHRA standard levels (including also ATDHRA as a small later eluting peak) in serum, brain and liver after control-treatment (CTRL, endogenous level, top chromatograms) and after 9CDHROL-supplementation (bottom chromatograms) in mice using the same *y*-axis scale (relative intensity in 303 -> 207 *m*/*z*) dimension per relevant organ (serum, brain, liver). After achieving the best sensitivity on the MS-MS detection mode at 303 -> 207 *m*/*z*, the presence of additional further co-eluting peaks with similar fragmentation pattern, like various dihydroretinoic acid-derivatives/acyclo-dihydroretinoic acid-derivatives and their geometric isomers, could be determined. We are currently in the process of carefully identifying these derivatives. Due to the high magnification of the *y*-axis the endogenous levels of 9CDHRA may be difficult to recognize. (**F**). Mnemonic performance of WT (*n* = 8) and RXRγ-/- (*n* = 5) mice was tested in DNMTP task at long ITIs in non-treated mice (NT, see text below) and following vehicle (VEH)-treatment or 40 mg/kg 9CDHROL-treatment. Data in graphs represent SEM. Statistical significance (Bonferroni post-hoc tests) is indicated in comparison with VEH group (*, *p* > 0.05), and in comparison, with WT mice treated with 9CDHROL (#, *p* > 0.05).

To address the efficiency of ATROL vs. 9CDHROL as potential substrates for generation of 9CDHRA, we have compared 9CDHRA levels in mouse serum, brain and liver following the corresponding animal treatments. ATROL treatment did not lead to any significant increase of ATRA levels as determined 11 h after treatment (Figure 3A), due to a complex homeostatic regulation reported previously [32,33]. It did not significantly alter 9CDHRA levels in the serum and only moderately increased its levels in the brain (ca. 6-fold) and in the liver (ca. 3-fold) (Figure 3C). In contrast, 9CDHROL appeared to be an excellent precursor of 9CDHRA (Figure 3B,C,E), as treatment with an equivalent amount of 9CDHROL led to about 100-fold increase of 9CDHRA in the serum and the liver, while ca. 1000-fold augmentation in the brain (Figure 3B,C). The high storage capacity of 9CDHROL and its conversion rates to 9CDHRA after 9CDHROL treatment are remarkable, whereas ATROL itself was neither well stored after ATROL treatment nor well metabolized to either 9CDHROL, 9CDHRA, or ATRA, which indicates a highly-controlled homeostatic regulation of ATROL level as well as its conversion to bioactive metabolites and hormonal ligands like ATRA and 9CDHRA (Figure 3D), or derivatives for further storage like retinyl esters which were not investigated in this study. We also found that the metabolic conversion of 9CDHROL to 9CDHRA can be cell-specific since treatment of oligodendrocytes with 10^−6^ M 9CDHROL led only to weak, but significant, increase of 9CDHRA (Figure 3B). In contrast, no increase of 9CDHRA was observed after equimolar treatment with ATROL (Figure 3B). The observation that 9CDHROL is a highly potent precursor of 9CDHRA in mouse, whereas ATROL, acting as vitamin A1, cannot or is only very weakly metabolized to 9CDHRA following administration of high dose ATROL, indicates that 9CDHROL can be considered as a new class of a vitamin or new type of the vitamin A family, which we named “Vitamin A5/X”, with 9CDHRA being its active form.

(€)—Inserted is an additional Figure with a specifically set y-axis for improved vision of relevant lower values; (*)—statistical significance in (**B**,**C**,**E**) in comparison to CTRL samples is indicated with a star when p < 0.05.

### 3.3. Vitamin A5/X-Specific Activity: RXR-Mediated Signaling Specific Activity of 9CDHROL 

To address the functional relevance of 9CDHROL as precursor of 9CDHRA for biological functions *in vivo* we have tested its pro-mnemonic activity in spatial working memory in delayed non-match to place (DNMTP), the task known to be sensitive to 9CDHRA activity [11]. Considering that RXRγ was previously shown to be the functionally predominant RXR in control of working memory and mnemonic effects of retinoids [11,15], we used mice carrying null mutation of this receptor (RXRγ-/-) to determine its involvement in the mediating memory effect of 9CDHROL. To this end we trained WT and RXRγ-/- mice in the T-maze using DNMTP protocol over 10 days to attain the criterion of optimal procedural performance measured by correct choices of rewarded arm (Supplemental Figure S1A). In the following days we determined limits of their mnemonic performance by identifying for each mouse the shortest delay between the acquisition and test trial (inter-trial-interval, ITI) at which the mouse performed at chance level (mean ITI = 10.5 min for WT and ITI = 3 min for RXRγ-/- mice; Supplemental Figure S1B). We found that acute treatment with 40 mg/kg of 9CDHROL significantly altered memory performance of tested mice, but such effect was dependent on genotype as indicated by significant interaction between treatment and genotype in two-way ANOVA analyses (F (1, 11) = 7234; *p* < 0.05). Post-hoc analyses using Bonferroni test revealed that such difference reflects significant improvement of memory performance in WT, but not RXRγ-/- mice. Accordingly, 9CDHROL-treated WT mice displayed significantly better performance attaining the mean of 74% of correct choices, which was significantly more than 54% scored for WT mice after vehicle treatment or 50% and 52% scored by RXRγ-/- treated with vehicle or 9CDHROL, respectively (Figure 3F). For comparison, in non-treated animals 46% correct choices were observed for WT-mice and 48% for RXRγ-/- mice with no significant difference compared to vehicle treated animals.

### 3.4. The Provitamin A5/X Concept: 9CDHBC Is a New Carotenoid of Plant Origin and an Efficient Precursor of 9CDHRA in Mice

**Figure 4:**
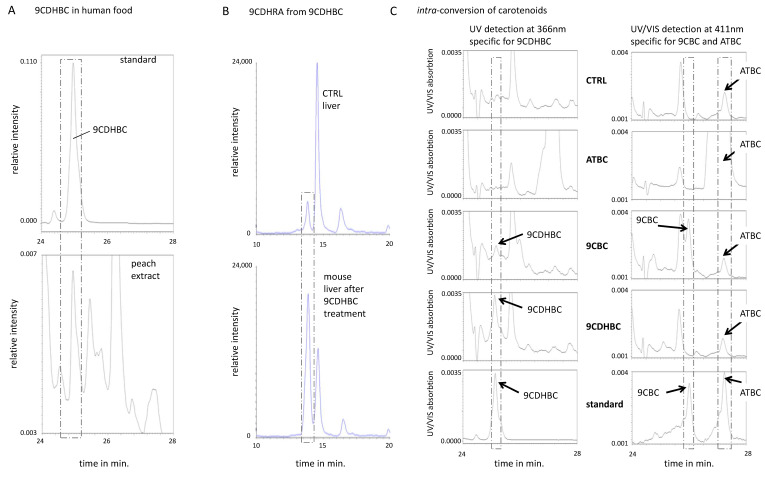
**The provitamin A5/X concept, 9CDHBC as a 9CDHRA precursor.** (**A**). 9CDHBC in the human food chain: standard of 9CDHBC eluting at 25.0 min (upper panel) and extract from canned peach (bottom panel) with a peak co-eluting at 25.0 min (indicated by a dashed line box) displayed with *y*-axis scale of relative intensity in 405 -> 95 *m*/*z* and a comparable UV/VIS spectrum (data not shown). (**B**). Conversion of 9CDHBC to 9CDHRA: 9CDHRA (indicated by a dashed line box) and ATDHRA (all-*trans*-dihydroretinoic acid) as peak eluting after 9CDHRA standard level after control-treatment (CTRL, physiological-relevant value, top chromatogram) and after 9CDHBC-supplementation (bottom chromatograms) in mouse liver (§). (**C**). Intra-conversion of carotenoids: left panel, indicating conversion of administered 9CBC to 9CDHBC in human oligodendrocyte cell line *in vitro.* Dashed line box indicates a peak with a retention time of 25.1 min of 9CDHBC standard and present after 9CDHBC treatment (fourth figure from the top at the left panel) as well in lower levels after 9CBC treatment (third figure from the top at the left panel), all detected at 366 nm and the same *Y*-axis. Right panel indicates no conversion of ATBC to either 9CBC or 9CDHBC in oligodendrocyte human cell line *in vitro*. ATBC is eluting at 27.2 min (detectable at 411 nm, while having an UV_max_ of 450 nm and indicated by a dashed line box and with the same *y*-axis) and present at very high levels after ATBC-treatment (second figure from the top at the right panel). 9CBC (indicated by a dashed line box) is present as a major shoulder peak after 9CBC-treatment, being non-detectable after CTRL or alternative treatments.

All-*trans*-β,β-carotene (ATBC) is considered as natural provitamin A1 of plant origin, as it can be cleaved at the C15=C15′ bond to generate all-*trans*-retinal (ATRAL), which in turn can be either oxidized to ATRA or reduced to ATROL [34]. In analogy with this pathway, we speculated that 9-*cis*-dihydro-β,β-carotene (9CDHBC) could exist and act as a precursor of 9CDHRA, fulfilling thus a role of provitamin A5/X. To test this hypothesis, we first obtained 9CDHBC by stereoselective chemical synthesis (Figure 1B). We next determined that 9CDHBC is present in the human food matrix of plant origin, as 9CDHBC standard was eluting at 25.0 min (Figure 4A, top panel) comparable with a corresponding peak in peaches extract with an estimated concentration of 77 ng/100 g (Figure 4A, bottom panel). Further parallel analytical proof of endogenous occurring 9CDHBC could not be performed because of the low endogenous levels. To address the possibility that 9CDHBC may act as provitamin A5, we performed *in vivo* supplementation studies with oral application of 9CDHBC in mice to test whether it could be converted to 9-*cis*-dihydroretinoids. A weak, but significant, 1.6-fold increase of 9CDHRA was observed in the liver of 9CDHBC treated mice (40 mg/kg bw) (Figure 3C and Figure 4B). Likewise, a significant increase was also noted in the brain (6-fold) and serum (2-fold) of 9CDHBC-supplemented mice (Figure 3C). We did not observe a significant increase of 9CDHROL levels in the serum, brain and liver (Figure 2B), thus suggesting that 9CDHBC might have been converted directly to 9CDHRA through central cleavage and further oxidation via the potential intermediate metabolite 9-*cis*-13,14-dihydroretinal.

*(§)—Y-axis scales displaying relative intensity in 303 -> 207 m/z were identical.*

In comparison to other carotenoids, 9CDHBC is a preferential precursor of 9CDHROL in cultured oligodendrocytes, since following 9CDHBC-treatment the level of 9CDHROL rose by ca. 7-fold (Figure 2F). In contrast, after ATBC-treatment we did not identify any detectable levels of 9CDHROL or 9CDHRA (Figure 2F and Figure 3B), whereas treatment with 9-*cis*-β-carotene (9CBC, synthesis shown in Figure 1C) led to only weak (ca. 4-fold) but significant increase of 9CDHROL levels (Figure 2F,G). The detection of 9CDHROL after 9CBC treatment may reflect indirect conversion of 9CBC to 9CDHROL via formation of 9CDHBC (Figure 2F,G), since the latter was detected in cultured oligodendrocytes after 9CBC treatment but neither after ATBC nor vehicle treatment (Figure 4C, left panel; 3rd row (9CBC), 2nd row (ATBC) and 1st row (vehicle)). 

### 3.5. Identification of 9-cis-13,14-Dihydroretinyl- and 9-cis-13,14-Dihydroretinoyl-Esters as Precursors for 9CDHRA in Cultured Oligodendrocytes

Retinyl esters are the major, safe, dietary source of vitamin A1 [20]. We therefore investigated whether 9-*cis*-dihydroretinyl-acetate (-ester / 9CDHROL-ES) or 9-*cis*-13,14-dihydroretinoyl-acetate (-esters / 9CDHRA-ES) can act as potential precursors of 9CDHROL and, more importantly, of 9CDHRA. Following targeted chemical synthesis of both esters (Figure 1A,B), we tested their conversion to the corresponding retinoids. We observed significant and strong increase of 9CDHROL and 9CDHRA levels 18 h after application of 10^−6^ M 9CDHROL-ES (Figure 2F and Figure 3B). Importantly, 9CDHRA-ES appeared to be an excellent precursor of 9CDHRA which increased ~2860-fold. Such high induction was not observed even after application of 9CDHRA itself (~500-fold). The level of 9CDHROL-ES remained elevated in 9CDHROL-ES treated cell even 18 h after treatment (~22-fold when compared to control conditions) suggesting a potential of this compound as a long acting source of 9CDHRA (Figure 3B).

## 4. Discussion

Here we discovered 9CDHROL as a new endogenous retinoid, with potential important physiological relevance and as a core member of a new class of vitamin or as a new independent member of the vitamin A family, named vitamin A5/X. It is found in vertebrates including mice and humans as well as in cattle, as a relevant example of the human food chain. 

9CDHROL is distinct from ATROL (vitamin A1-alcohol) in several aspects. Most importantly 9CDHROL, but not ATROL, is efficiently metabolized to 9CDHRA, which was recently identified as an RXR ligand with physiological relevance, which may directly mediate RXR-specific pro-mnemonic activities. Despite such differences, we cannot exclude minor indirect metabolic links between vitamin A1 and A5/X pathways. In mice using moderate to high supplementation doses of either retinoid (ATROL or 9CDHROL), such a conversion was not observed in the three representative compartments of the organism analyzed in this study. However, oligodendrocytes cultured *in vitro* displayed weak, but significant, conversion of ATROL to 9CDHROL following treatment with a high concentration of ATROL, likely due to an optimal 9-*cis* specific metabolic environment for direct enabling of RXRγ-mediated signaling shown previously to be important for remyelination process [35]. Finally, in contrast with the high homeostatic regulation of vitamin A1 at various levels [32,33,34], vitamin A5/X bioavailability in the organism seems to be regulated more in a food intake and specific nutrient-dependent switch to enable RXR-mediated signaling. 

**Figure 5:**
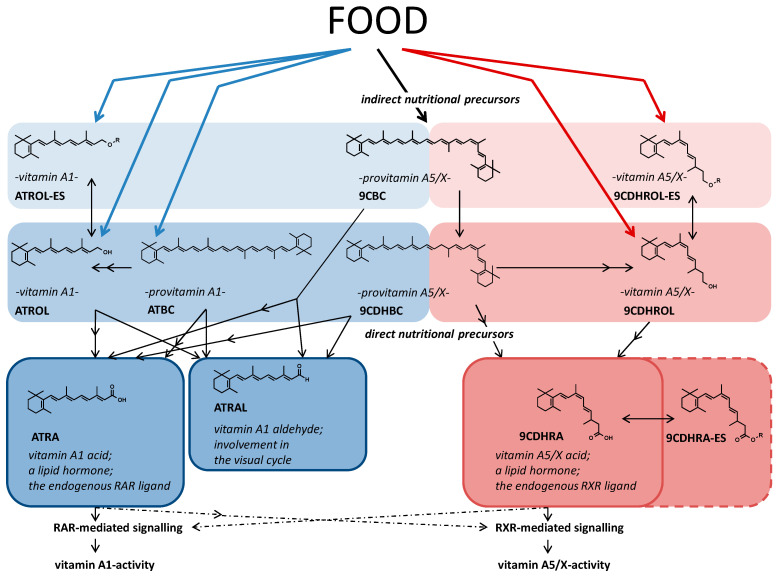
**Metabolic pathway of the novel vitamin A5/X / provitamin A5/X pathway.** Simplified metabolic pathway including structural formula starting from nutritionally derived carotenoids and retinoids and key metabolites of the vitamin A1 cluster pathway on the left side and colored in blue background: all-*trans*-retinyl ester (ATROL-ES), all-*trans*-retinol (ATROL), all-*trans*-β,β-carotene (ATBC), 9-*cis*-β,β-carotene (9CBC) to all-*trans*-retinal (ATRAL) and all-*trans*-retinoic acid (ATRA) as the bioactive metabolites of the vitamin A1 cluster pathway. On the right side, and colored in reddish background, the novel claimed vitamin A5/X cluster pathway with 9CBC as a partial vitamin A1 / A5/X derivative representing the indirect nutritional vitamin A5/X precursor, with 9-*cis*-13,14-dihydroretinyl esters (9CDHROL-ES) as well as the direct nutritional precursor vitamin A5/X alcohol 9-*cis*-13,14-dihydroretinol (9CDHROL) and the provitamin A5/X carotenoid 9-*cis*-13,14-dihydro-β,β-carotene (9CDHBC), which can be transformed to the lipid hormone - vitamin A5/X acid 9-*cis*-13,14-dihydroretinoic acid (9CDHRA) as the resulting bioactive and endogenous RXR-ligand. In addition, direct 9CDHRA-precursors 9-*cis*-13,14-dihydroretinoyl ester (9CDHRA-ES) were still not endogenously identified.

We also show that the new carotenoid 9CDHBC acts as an efficient provitamin A5/X compound, is also found in the food matrix of plant origin, shown here as an example in peach extracts, and may act as a proximate intermediate in the biosynthesis of 9CDHRA (Figure 5). Whereas ATBC acts as an efficient provitamin A1 source, it is not metabolized *in vivo* (mice) and *in vitro* (oligodendrocytes) to 9CDHROL or 9CBC, and further to 9CDHRA. Instead, 9CBC is present in humans with preferred accumulation in organs, while less abundant in the serum [36] and moderately abundant in the human food chain, and may represent the nutritionally-relevant provitamin A5/X (present data and [37,38,39]). 9CBC originates mainly from plant-based metabolism [40] or from food processing of ATBC [38,41]. As mice are not an optimal model to study nutri-kinetics of carotenoids [42], we speculate a more likely scenario of 9CBC obtaining an important nutritional-relevant precursor function in humans. This more important nutritional relevance to humans can additionally be speculated due to its weak-moderate precursor potential for 9CDHBC in directly exposed *in vitro* cultured oligodendrocytes.

Future studies of this cluster of compounds should contribute to understanding the pleiotropic activities of RXRs, and especially the physiological function of the RXR-ligand and its nutritional precursors. RXR-mediated signaling, which coordinates signaling pathways with several heterodimer binding partner nuclear receptors like PPARs, LXR and NR4A as permissive and RARs and vitamin D receptor (VDR) as non-permissive partners [4,43], is selectively augmented in various neurological diseases: obesity, diabetes and further diseases of the cardiovascular system such as atherosclerosis [1,2,5]. Further studies should focus on details of the metabolic pathway of these food derived compounds like provitamin A5/X (9CBC / 9CDHBC) involving BCO1/2-mediated cleavage pathways [44,45,46,47], as well as starting from the food precursor vitamin A5/X (9CDHROL / 9CDHROL-ES) towards the bioactive RXR-ligand 9CDHRA via a further ALDH1A-oxidation [48,49,50,51]. In consequence, vitamin A5/X and provitamin A5/X might be an important nutritional constituent identified in the human organism and human food chain to prevent such common Western-lifestyle based diseases. As an example, memory functions representing memory impairment as a selective RXR-mediated signaling dysfunction were used in this study [14,15,27,35,52,53] and we determined a pro-mnemonic activity of 9CDHRA [11] as well as 9CDHROL. 

We anticipate that enhancement/modulation of general vitamin A deficiency syndromes in humans by selectively modulating the vitamin A5/X signaling pathway instead of vitamin A1 signaling pathways via treatment with vitamin A5/X cluster derivatives may be selectively beneficial for the treatment of dysfunctions with a major RXR-involvement and RXR-dysfunctionality as determined in RXR-mediated signaling dependent diseases and various neurological diseases such as psychotic diseases, dysfunctional myelination diseases and neurodegenerative diseases as well as cancer, atherosclerosis, allergies and obesity/diabetes [1,2,5].

## 5. Conclusions

Based on the summed up criteria, we have determined a novel vitamin A1-independent pathway of proximate precursors like 9CDHROL, 9CDHBC and the further upstream precursor 9CBC. These food derived precursors provide sufficient endogenous and nutritional relevant levels of the physiological endogenous RXR ligand 9CDHRA to further enable physiologically crucial RXR-mediated signaling starting from the diet; we named this the “vitamin A5/X pathway”.

## Data Availability

The datasets analysed in the present study are available from the corresponding authors.

## Supplementary Materials

The following are available online at https://www.mdpi.com/2072-6643/13/3/925/s1, Figure S1: (**A**) Performance of WT and RXRγ-/- mice during procedural memory acquisition in the delayed non-match to place task in the T-maze. The total of *n* = 8 WT and *n* = 5 RXRγ-/- mice acquired the delayed task over 10 consecutive days of training as indicated by the main effect of training day (F (9, 99) = 13,28; *p* < 0.001) and the evolution of the acquisition curve was comparable between WT and RXRγ-/- mice as there was no significant interaction between genotype and training day (F (9, 99) = 0,6; *p* = 0.8). However, there was a significant difference of genotype as indicated by the main group effect for this variable (F (1, 11) = 19,47; *p* < 0.01), reflecting an overall lower performance of RXRγ-/- mice, although post-hoc analyses using Bonferroni test did not reveal a difference for any specific time point. (**B**) The Inter-Trial-Interval (ITI) test, used for testing mnemonic effects of 9CDHROL treatment, identified for each individual mouse as the minimum time at which it performed at 60% of correct choices or less. Two-tailed t-test revealed only a tendency for statistical difference in test ITI (t = 2193, *p* = 0.051).

## Abbreviations

all-*trans*-retinol—ATROL, all-*trans*-β,β-carotene—ATBC, all-*trans*-retinoic acid—ATRA, all-*trans*-retinal—ATRAL, 9-*cis*-13,14-dihydroretinol—9CDHROL, 9-*cis*-13,14-dihydroretinoic acid—9CDHRA, 9-*cis*-β,β-carotene—9CBC, 9-*cis*-13,14-dihydro-β,β-carotene—9CDHBC, 9-*cis*-dihydroretinyl-ester—9CDHROL-ES, 9-*cis*-dihydroretinoyl-ester—9CDHRA-ES, retinoid X receptors—RXR, liver X receptor—LXR, nuclear receptor subfamily 4 group A (NR4A), peroxisome proliferator-activated receptor—PPAR, delayed non-match to place—DNMTP, vitamin D receptor—VDR.
