# Supplementary material for: Vitamin A5/X, a New Food to Lipid Hormone Concept for a Nutritional Ligand to Control RXR-Mediated Signaling
Source: Nutrients. 2021 Mar 12;13(3):925. doi: 10.3390/nu13030925 (PMC7999121; doi:10.3390/nu13030925)

Supplementary Figure 1.

a) Performance of WT and RXRγ-/- mice during procedural memory acquisition in the delayed non-match to place task in the T-maze. The total of n=8 WT and n=5 RXRγ-/- mice acquired the delayed task over 10 consecutive days of training as indicated by the main effect of training day (F (9, 99) = 13,28; p<0.001) and the evolution of the acquisition curve was comparable between WT and RXRγ-/- mice as there was not significant interaction between genotype and training day (F (9, 99) = 0,6; p=0.8). However, there was a significant difference of genotype as indicated by the main group effect for this variable (F (1, 11) = 19,47; p<0.01) and reflecting an overall lower performance of RXRγ-/- mice, although post-hoc analyses using Bonferroni test did not reveal difference for any specific time point.

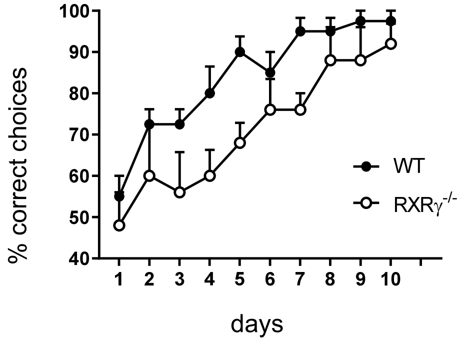

b) The test Inter-Trial-Interval (ITI) used for testing mnemonic effects of 9CDHROL treatment was identified for each individual mouse as the minimum time at which mouse performed at 60% of correct choices or less. Two-tailed t-test revealed only a tendency for statistical difference in test ITI (t=2,193, p=0.051).

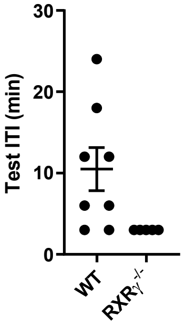

Supplement: Supplementary file 1 [file nutrients-13-00925-s001.pdf]
